# Feasibility study of a self-guided internet-based intervention for family caregivers of patients with cancer (OAse)

**DOI:** 10.1038/s41598-022-21157-9

**Published:** 2022-10-06

**Authors:** Miriam Grapp, Johanna Ell, Senta Kiermeier, Markus W. Haun, Andrea Kübler, Hans-Christoph Friederich, Imad Maatouk

**Affiliations:** 1 Department of General Internal and Psychosomatic Medicine, University Clinic Heidelberg, Im Neuenheimer Feld 410, 69120 Heidelberg, Germany; 2 National Center for Tumor Diseases Heidelberg, University Clinic Heidelberg, Im Neuenheimer Feld 460, 69120 Heidelberg, Germany; 3 Department of Psychiatry and Psychotherapy, University Medical Center Freiburg, Hauptstraße 6, 79106 Freiburg, Germany; 4 Section of Psychosomatic Medicine, Psychotherapy and Psychooncology, Department of Internal Medicine II, Julius-Maximilian University, Oberdürrbacher Straße 6, 97080 Würzburg, Germany; 5 Department of Psychology I, Institute for Psychology, Julius-Maximilian University, Marcusstraße 9-11, 97070 Würzburg, Germany

**Keywords:** Cancer, Psychology, Oncology

## Abstract

Despite high levels of distress, family caregivers of patients with cancer rarely seek psychosocial support and Internet-based interventions (IBIs) are a promising approach to reduce some access barriers. Therefore, we developed a self-guided IBI for family caregivers of patients with cancer (OAse), which, in addition to patients' spouses, also addresses other family members (e.g., adult children, parents). This study aimed to determine the feasibility of OAse (recruitment, dropout, adherence, participant satisfaction). Secondary outcomes were caregivers’ self-efficacy, emotional state, and supportive care needs. N = 41 family caregivers participated in the study (female: 65%), mostly spouses (71%), followed by children (20%), parents (7%), and friends (2%). Recruitment (47%), retention (68%), and adherence rates (76% completed at least 4 of 6 lessons) support the feasibility of OAse. Overall, the results showed a high degree of overall participant satisfaction (96%). There were no significant pre-post differences in secondary outcome criteria, but a trend toward improvement in managing difficult interactions/emotions (*p* = .06) and depression/anxiety (*p* = .06). Although the efficacy of the intervention remains to be investigated, our results suggest that OAse can be well implemented in caregivers’ daily lives and has the potential to improve family caregivers’ coping strategies.

## Introduction

Family or informal caregivers of patients with cancer must balance two roles: on the one hand, they are confronted with considerable life changes while dealing with their own, often unsettling, emotional responses to the patient’s diagnosis. On the other hand, many spouses and other close relatives of patients with cancer provide extensive and continuous support on instrumental, informational, and emotional levels^1,2^ and thus represent a fundamental resource for patients coping with their diseases^3,4^. Family caregivers are commonly directly involved in illness trajectories, and they undertake nursing tasks for which they are not trained, often feeling overwhelmed^5^.

“Caregiver-burden” is an established term both in cancer research and in clinical practice and is defined as “the extent to which family caregivers perceive that caregiving has an adverse effect on their emotional, social, financial, physical, and spiritual functioning”^6^. Several studies indicate that family caregivers of cancer patients have mental health issues^7,8^. Compared with the general population, these caregivers have increased distress, anxiety, and depression levels, and the prevalence of anxiety disorders and depression among family caregivers is markedly elevated^9,10^. In addition to fear, grief, and stress, family caregivers also report feeling anger about the situation^11^ and feelings of guilt toward the patients^12^. Despite these high stress levels, family caregivers of patients with cancer seek professional psychosocial support considerably less often than patients with cancer^13,14^. The low uptake is mainly due to tangible factors, such as lack of time since family caregivers often work and attend to their families simultaneously^15,16^. Other barriers include the lack of knowledge about existing services for family members^17^ and the fear of social stigmatization^14,18^. Family caregivers often also state that they do not need support or that they receive support from nonprofessionals (e.g., chaplaincy)^19^.

Internet-based interventions (IBIs) may overcome some of the above barriers and could be a promising approach to provide facilitated access to psychosocial support for family caregivers. They offer a high degree of flexibility in terms of time and place, they are also accessible for people living in rural areas^20^.

Over the past ten years, a growing number of IBIs have been developed in the field of psychooncology. However, they are primarily for cancer patients^21,22^; only recently, their focus has widened to include support for family caregivers with special burdens and needs^23^. Some IBIs, which also involve partners of cancer patients, are interventions for couples, focusing on communication and symptom management^24,25^. Additionally, there are online services for family caregivers, which are offered as social support group interventions. Some of them are combined with face-to-face sessions^26,27^. However, to the best of our knowledge, only few structured self-help Internet interventions explicitly target family caregivers^28–31^. These interventions are primarily aimed at the spouses of cancer patients. In German-speaking countries there is still a substantial lack of IBIs for family caregivers of patients with cancer and research in this field is in its very early stages so statements about the effectiveness of existing IBIs for family caregivers are still limited^32^.

Therefore, we developed a self-guided Internet-based intervention for family caregivers of patients with cancer (OAse). The acronym OAse stands for the German term *‘****O****nline-Unterstützungsangebot für ****A****ngehörige kreb****s****kranker Pati****e****nten’* (online intervention for family caregivers of patients with cancer). The intervention is intended to support family caregivers of patients with cancer in dealing with the psychosocial consequences of cancer, coping with their own burdensome feelings, and activating their own resources. OAse is in German and addresses not only the spouses of patients but also their adult children, parents, other family members, or peers. OAse is a resource-oriented, self-management program based on the concept of “Cancer Family Caregiving Experience”^12^ an updated and expanded conceptual model that comprehensively describes the situation of family caregivers of patients with cancer. This model entails three main elements: (1) the stress process (e.g., patient illness experiences, caregiving demands, relationships, finances, emotional impact, fatigue, and sleep), (2) contextual factors (personal, situational, and social contexts in which the stress process is entrenched, including personal and social characteristics, personality features, social support, and relationship quality), and (3) the cancer trajectory (the course of the disease process and treatment over time). The second theoretical pillar of OAse is the concept of “Salutogenesis”, which, according to Antonovsky^33^ addresses the question of how an individual remains healthy despite high stress levels and what resources might contribute to preserving health.

As described family caregivers are often unaware of their own needs and therefore, partners seek support for themselves. However, many caregivers are looking for further information and support in dealing with the distressing situation. Therefore, we have designed an easily accessible, module-based self-guided intervention, so that caregivers can engage with the offered content independently, flexibly and according to their own personal and temporal resources. For a detailed description of the development and structure of the intervention see the methods section under ‘intervention development’. The present study aimed to investigate the OAse intervention’s feasibility and acceptability before conducting an anticipated effectiveness trial.

## Methods

### Study design and participants

We conducted a feasibility study with a one-group pretest–posttest design. The intervention ran from August 2018 to November 2019. The study was conducted in accordance with the Declaration of Helsinki, and the Ethics Committee of the University of Heidelberg (S-196/2018) granted ethical approval. Family caregivers of adult patients with cancer undergoing curative or palliative oncological treatment were eligible to participate in this study. According to the American Cancer Society’s (ACS) National Quality of Life Survey for Caregivers (NQOL-CG), we defined a family caregiver as a “family-like” individual, nominated by the patient, and the one individual providing consistent help^34^. Their participation in the study was independent of the patients’ tumor sites and treatment stages. Further inclusion criteria were age 18 years and older, having Internet access and an adequate Internet-enabled device, and sufficient knowledge of the German language.

Family caregivers with a cancer diagnosis, with severe psychological comorbidity (such as substance abuse/dependence likely to compromise intervention adherence, risk of endangerment to others, and/or risk of self-endangerment or acute psychotic symptoms, e.g., persecutory delusions and/or thought insertion) or with severe cognitive or physical impairment that make using the intervention difficult were excluded from this study. We did not exclude the possibility of more than one family caregivers per patient participating in the intervention. However, the relationship to the patient was carefully recorded so that we could control for the participation of multiple caregivers of a patient.

### Recruitment and procedures

From June 2018 to March 2019, family caregivers of cancer patients were informed about the study via flyers, posters, and the website of the National Center for Tumor Diseases (NCT) in Heidelberg, Germany. Additionally, eligible family caregivers who accompanied patients to the NCT were personally approached by study team members. No specific enrollment goal was set, instead the number of family caregivers who could be enrolled in the study during the defined recruitment period of 10 months was recorded. Family caregivers who agreed to participate in the study were informed of the study goals and procedures. Informed consent was obtained from all participants. After completing a pre-treatment questionnaire (T_0_), the family caregivers received an email with access to the OAse intervention. After completing the intervention (or dropping out), the family caregivers participated in a post-treatment survey (T_1_). The family caregivers did not receive any financial compensation for their participation in the study.

### Intervention development

The development of OAse is based on the intervention mapping approach of Eldredge et al.^35^. In a first step, based on a systematic literature review, a problem analysis was conducted with focus on the particular burdens of family caregivers and their wishes/expectations with regard to online interventions. Based on this, the main topics were identified and appropriate theoretical models were selected. For OAse, as described in the introduction, these were the concepts of “Cancer Family Caregiving Experience”^12^ and “Salutogenesis”^33^. Then, a first version of the intervention was developed following this theoretical basis and the specification of the content. This pilot version was tested by five psychooncologists and four family caregivers for being appealing, containing intuitive functions, being easy to navigate, and providing understandable and meaningful information. The usability testers should mainly evaluate and provide feedback on the following aspects: (1) visual design (impression of the appearance of the intervention), (2) language and expression (e.g., whether the text is understandable and logical), (3) structure and content of the intervention (e.g., whether the content of each lesson is understandable, comprehensible, and appropriate), (4) functionality (interactive and adaptive features of the intervention), and (5) motivation to use (e.g., whether OAse is appealing to users).

**Figure 1:**
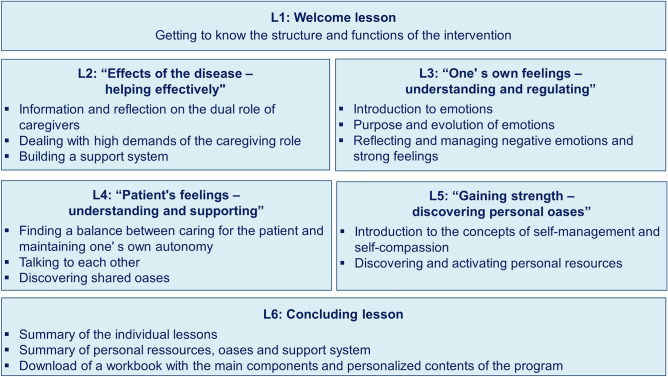
Overview of the structure and content of the OAse intervention. Shown are the title of the individual lessons with their respective thematic focus.

OAse was designed as an easily accessible, module-based intervention. The intervention should both cover the relevant topics and be oriented to the limited time of family caregivers. This resulted in the total number of 6 lessons with a completion time of about one hour per lesson. The lessons could be paused at any time and continued later (e.g., the following day). No specific time frame was given for the completion of the individual lessons or the total intervention. The participants were recommended to complete one lesson per week, which corresponds to a total completion time of 6 weeks. For an overview of the content and structure of OAse, see Fig. 1 and two additional screenshots from the intervention are provided in Supplementary Fig. S1.

OAse comprises psycho-educative elements, exercises for self-reflection and skill-building, mindfulness and guided imagination exercises, and experiences of other (fictitious) caregivers guiding the participants through the intervention (model caregivers). The psychoeducational elements with the aim of providing information for a better understanding of the respective situation were presented as text, pictures and flow-charts. The model caregivers are introduced in the welcome lesson with a picture and their individual story and accompanied the participants through all lessons in form of their own testimonials, presented as written text. The exercises for self-reflections and skill-building are active program components in which patients can engage more intensively with the contents of the individual lessons and implement them in everyday life. The exercises are recommended in this program, but are not a requirement for completing the following lessons. The guided imagination exercises are presented as audio files and can be downloaded by the participants to be used at any time.

OAse is designed as self-guided intervention; therefore, there was no therapeutic support in terms of regularly individualized feedback. However, the participants had the opportunity for personal contact with a clinical psychologist and research team member via the chat function within the program or during telephone consultations (1 h per week). The telephone consultations were available every week at a specific time in the late afternoon, so that it was also suitable for working participants. If participants were not able to seek contact within this time slot, they could request an individual appointment via the chat function if needed. At the end of each lesson, the intervention also included important information about whom participants could contact in case of emergency and severe psychological distress. OAse is not designed as dyadic or shared intervention; each participating relative worked through the intervention on their own.

### Measures

#### Sample characteristics

Sociodemographic information of family caregivers (cis-/transgender, age, household, and employment status), the patients’ clinical characteristics (cancer diagnosis, time since diagnosis and metastasized cancer (yes/no), and kind of treatment), and the relationships between patients and family caregivers were recorded using a baseline questionnaire at T_0_.

#### Primary outcomes: feasibility and acceptability

Feasibility and acceptability were assessed in terms of recruitment, attrition, adherence, and participant satisfaction with OAse. Recruitment was operationalized as recruitment method (flyer, poster, website, or personal approach) and number of family caregivers approached that were enrolled in the study. Attrition was measured by the number of family caregivers who dropped out before completing the program. Adherence referred to the extent to which the family caregivers engaged with the web-based intervention and was operationalized as the number of lessons completed and the extent to which the participants used the additional OAse elements. Participant satisfaction with OAse was assessed post-intervention (T_1_) via an author-generated questionnaire containing 12 Likert-type scales. The overall satisfaction with OAse was assessed with the item ‘overall, I am satisfied with OAse’ and a total of additional 11 study specific items covered different aspects of user satisfaction regarding the intervention’s form and the content, the program’s subjective and personal benefits, and the practicability of the intervention and integration into daily living. Participants answered these items on a 5-point scale with the response options ‘strongly agree’, ‘agree’, ‘partially agree’, ‘disagree’, and ‘strongly disagree’. The questionnaire was supplemented with three open-ended questions, eliciting information about the elements of the intervention family caregivers found particularly helpful, perceived barriers to using OAse, and the suggestions for improvement. Thematic analysis^36^ was also employed to analyze open-ended questions.

#### Secondary outcomes

At baseline (T_0_) and post-intervention (T_1_), the following validated questionnaires were administered: the perceived general self-efficacy of the family caregivers was assessed with the General Self-Efficacy Scale (GSES)^37^. The GSES is a 10-item questionnaire measuring the strength of the general conviction of individuals that they can effectively cope with difficult situations and obstacles. It is considered as reliable and valid instrument for assessing general self-efficacy (Cronbach's *α* = 0.80–0.90). The Caregiver Inventory (CGI)^38^ was used as an additional measure of self-efficacy for caregiving. The CGI comprises 21 items and was designed to assess caregiving self-efficacy expectations of caregivers, who were providing informal caregiving. The questionnaire has excellent internal consistency (Cronbachs' *α* = 0.91). Additionally, based on a factor analysis of the original CGI, four subscales were derived (1) managing medical information (Cronbachs' *α* = 0.64), (2) caring for the care recipient (Cronbachs' *α* = 0.78), (3) caring for oneself (Cronbachs' *α* = 0.88), and (4) managing difficult interactions/emotions (Cronbachs' *α* = 0.76). For this study, only two dimensions, namely, caring for oneself (five items) and managing difficult interactions and emotions (six items), were used, which were previously translated into German with the kind permission of the authors. The family caregivers’ temporary emotional state was evaluated with the German short version of the Profile of Mood States (SV-POMS-D)^39^. The SV-POMS-D is a 35-item questionnaire that measures mood and emotional distress over a period of time (the past 24 h) on four different scales: depression/anxiety, fatigue, vigor, and hostility. The internal consistency of the questionnaire is high (Cronbach's *α* = 0.88-0.95) and its factorial validity is considered satisfactory. Additionally, at T_0_, the multi-dimensional supportive care needs of family caregivers were measured with the German version of the Supportive Care Needs Survey for Partners and Caregivers (SCNS-P&C-G)^10^. The SCNS-P&C-G is a 45-item self-report questionnaire that measures family caregivers’ needs in four domains: health-care service needs, psychological and emotional needs, work and social needs, and information needs. The internal consistency for each dimension is high (Cronbach's *α* = 0.76-0.95) and convergent validity is classified as high.

### Data analysis

Statistical analyses were performed with IBM SPSS (Version 25) software. Descriptive statistics were obtained for the family caregivers’ sociodemographic variables; for the patients’ clinical characteristics; for family caregivers’ supportive care needs (SCNS-P&C-G); and for attrition, adherence, and participant satisfaction. The reasons for attrition were also collected, and the group differences between completers versus non-completers were analyzed using Mann–Whitney U tests for continuous variables and χ2 tests of independence and the Fisher exact test for categorical variables. The sample was not powered to detect significance in the secondary outcome measures. Nevertheless, we presented non-parametric data (Wilcoxon signed-rank test) in relation to self-efficacy (GESE and CGI), and emotional distress (SV-POMS-D) to help clarify the potential effect of the intervention within this sample and to provide data on which a power calculation for a larger study of efficacy can be based. The Bonferroni–Holm method^40^ was also employed to counteract the problem of multiple comparisons, and the effect size for the Wilcoxon signed-rank test was calculated on the basis of correlation coefficient r according to the formula r = Z/√N^41^.

## Results

### Sample characteristics

From June 2018 to April 2019, n = 41 family caregivers of patients with cancer were included in the study. Of the 41 family caregivers, 66% (n = 27) were female. The participants ranged in age from 19 to 69 years (M = 44.59, SD = 13.75). Most of the participants were spouses (n = 29, 71%), followed by adult children (n = 8, 20%), parents of adult patients (n = 3, 7.3%), and friends (n = 1, 2.4%). Although it had been possible according to the inclusion criteria, no multiple caregivers for a single patient participated. A total of 81% (n = 33) of the participants shared a common household with the patients, and 73% (n = 30) of the participants were employed with 66% (n = 27) working more than 20 h per week. About half of the patients had metastatic tumor diseases (n = 19, 46%), and the time since diagnosis ranged from 1 to 73 months (M = 12.85, SD = 18.25). There was a considerable spectrum of tumor entities, but the most common cancer sites were the breast (n = 10, 25%), skin (n = 8, 20%), ovary (n = 5, 13%), and pancreas (n = 4, 11%). Slightly more than half of the patients received chemotherapy (n = 24, 59%), a small number received radiotherapy or immunotherapy (n = 3, 7.3% each), and 27% (n = 11) of them did not undergo treatment. All participants owned at least one Internet-ready devices and n = 32 (78%) reported using the Internet several times a day, n = 6 (15%) used the Internet once a day, and n = 3 (7.3%) used the Internet at least once a week. N = 2 (4.9%) participants had previously participated in a web-based health intervention (e.g., web-based relaxation training).

### Primary outcomes

#### Recruitment, attrition and adherence

N = 13 participants were recruited via flyers (n = 3), posters (n = 2), and the website of the National Center for Tumor Diseases (NCT) in Heidelberg (n = 8). In addition, a total of n = 60 family caregivers were approached personally by the study team. Of these, n = 28 (47%) agreed to participate in the study and were included. The other n = 32 (53%) caregivers approached declined to participate in the study for the following reasons: no time (n = 11), no need (n = 9), no interest in / reservations about online format (n = 7), already existing psycho-oncological support (n = 2), no computer, smartphone, tablet, etc. available (n = 2), and do not consider themselves a close relative (n = 1).

A total of 41 family caregivers were included in the study, of whom n = 28 (68%) completed the full OAse intervention and n = 3 caregivers (7.3%) never started. N = 3 (7.3%) completed one, n = 1 (2.4%) two, n = 3 (7.3%) three, n = 2 (4.9%) four, and n = 1 (2.4%) five of the six lessons. Which means, n = 31 caregivers (75%) carried out a substantial part of the intervention (at least 4 out of 6 lessons) and each caregiver worked on average 4.75 out of 6 lessons.

The completion time for the full intervention (T_0_ to T_1_) ranged from 4 to 12 weeks and was on average 7.5 weeks (SD = 4.1 weeks). The reasons reported for dropping out were technical difficulties (n = 4, 9%), the lack of time (n = 3, 7.3%), patient deceased during participation (n = 2, 4.9%), dissatisfaction with the intervention content (n = 2, 4.9%), and not specified (n = 2, 4.9%). We compared the demographic and disease characteristics and the baseline scores of family caregivers who completed the full intervention (“completers”) with family caregivers who dropped out before completing the intervention (“non-completers”) (Table 1). There were no differences within the sociodemographic and disease-related variables. Moreover, we found no differences between completers and non-completers regarding the SWE, CGI, POMS, and SCNS-SF34 baseline scores.

**Table 1:** Participants’ sociodemographic characteristics, patients’ disease characteristics, and baseline questionnaire scores of completers and non-completers.

	Participants (n = 41)	Completers (n = 28)	Non-completers (n = 13)	*P*
Age in years (mean, SD)	44.6 (13.7)	45.3 (10.7)	39.0 (13.8)	0.33
**Sex (n, %)**				
Male	14 (34%)	10 (36%)	4 (31%)	0.99
Female	27 (66%)	18 (64%)	9 (69%)	
**Relationship with patients (n, %)**				
Spouse	29 (71%)	22 (79%)	7 (54%)	0.21
Child	8 (20%)	4 (14%)	4 (31%)	
Parents	3 (7.3%)	1 (3.6%)	2 (15%)	
Friend	1 (2.4%)	1 (3.6%)	–	
**Common household (n, %)**				
Yes	33 (80%)	25 (89%)	8 (62%)	0.08
No	8 (20%)	3 (11%)	5 (38%)	
**Occupation (n, %)**				
Yes	30 (73%)	21 (75%)	9 (69%)	0.72
No	11 (27%)	7 (25%)	4 (31%)	
Time since diagnosis (mean, SD)	12.8 (18.3)	9.8 (13.5)	19.9 (25.6)	0.41
**Metastasized (n, %)**				
Yes	19 (46%)	12 (44%)	7 (54%)	0.50
No	20 (49%)	15 (54%)	5 (38%)	
Missing	2 (4.9%)	2 (7.1%)	1 (7.7%)	
**Kind of treatment (n, %)**				
Chemotherapy	24 (59%)	16 (57%)	8 (62%)	0.43
Radiotherapy	3 (7.3%)	1 (3.6%)	2 (15%)	
Immunotherapy	3 (7.3%)	3 (11%)	–	
No treatment	11 (27%)	8 (28%)	3 (23%)	
GSES score (mean, SD)	28.0 (4.0)	28.3 (3.9)	28.6 (3.6)	0.93
**CGI (mean, SD)**				
Subscale caring for oneself	28.7 (6.3)	28.8 (6.8)	28.4 (8.4)	0.55
Subscale managing difficult interactions and emotions	36.1 (7.5)	36.1 (8.32)	36.25 (7.9)	0.68
**SV-POMS-D (mean, SD)**				
Subscale depression/anxiety	27.5 (16.5)	28.5 (17.4)	22.6 (14.9)	0.57
Subscale fatigue	20.3 (10.4)	20.4 (10.5)	18.9 (12.3)	0.81
Subscale hostility	13.5 (10.6)	12.8 (11.8)	10.4 (9.2)	0.96
Subscale vigor	18.9 (7.5)	20.8 (8.6)	17.6 (5.4)	0.20
SCNS-P&C-G score (mean, SD)	91.4 (33.5)	92.7 (35.7)	74.8 (26.8)	0.87

*GESE* General Self-Efficacy Scale, *CGI* Caregiver Inventory, *SV-POMS-D* German short version of the Profile of Mood States, *SCNS-P&C-G* German version of the Supportive Care Needs Survey for Partners and Caregivers.

*P*-values from the Chi-square test, Fisher’s exact test, and Mann–Whitney *U* test.

The OAse elements self-reflection and mindfulness and guided imagination exercises were used by the majority of the participants (n = 37, 87% and n = 33, 82%, respectively). N = 10 (24%) of the participants regularly communicated via the integrated chat function, whereas only n = 3 (7.3%) used the telephone consultation. Participants using the chat function or telephone consultation did not differ from the overall sample in any characteristic (e.g., age, gender, relationship to patient).

The OAse elements self-reflection and mindfulness and guided imagination exercises were used by the majority of the participants (n = 37, 87% and n = 33, 82%, respectively). N = 10 (24%) of the participants regularly communicated via the integrated chat function, whereas only n = 3 (7.3%) used the telephone consultation. Participants using the chat function or telephone consultation did not differ from the overall sample in any characteristic (e.g., age, gender, relationship to patient).

#### Participant satisfaction

**Figure 2:**
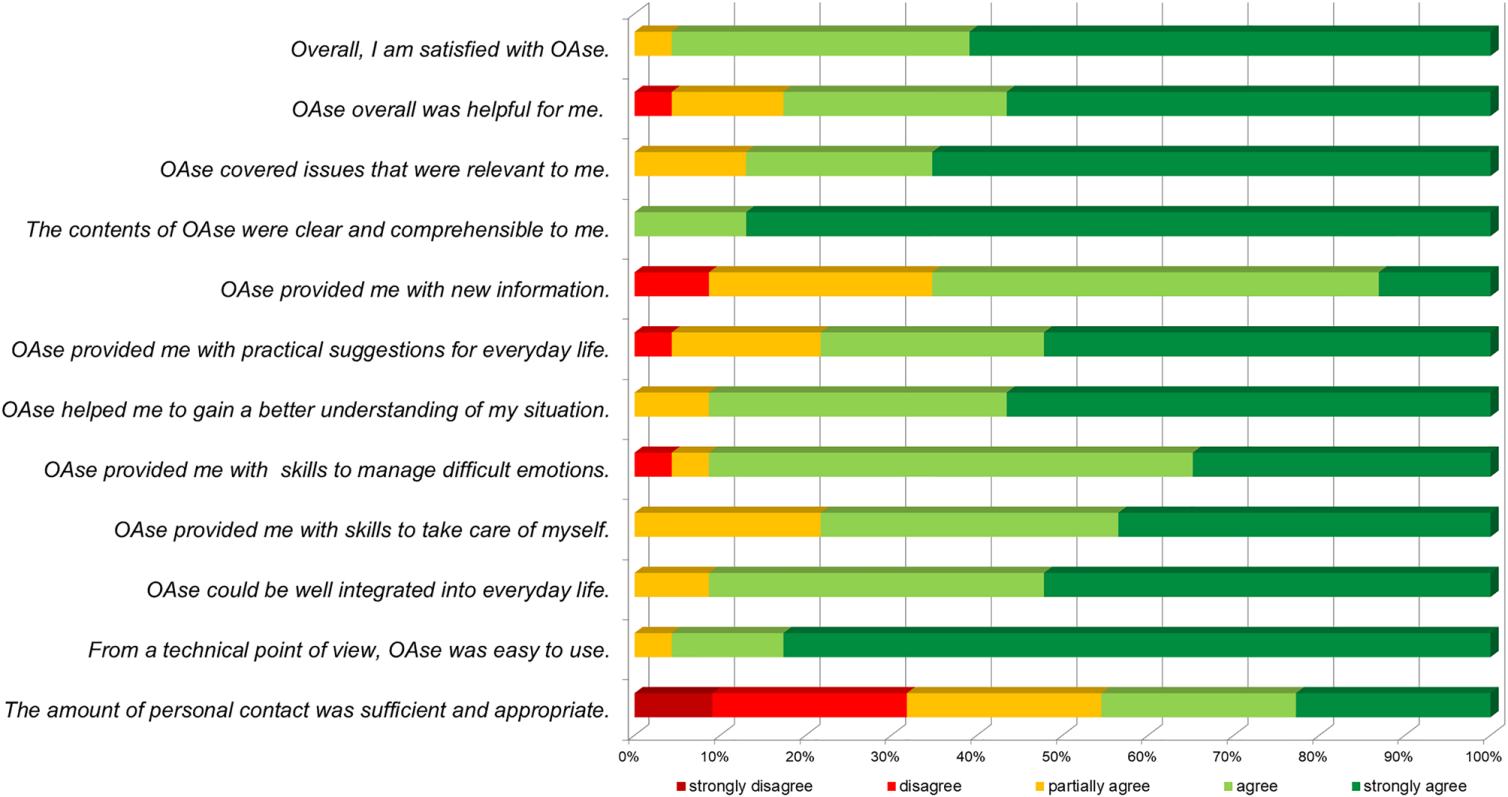
Participant satisfaction with OAse. Shown are the results of the author-generated questionnaire containing 12 Likert-type scales on the following dimensions: satisfaction with the intervention’s form and the content, the program’s subjective and personal benefits, and the practicability of the intervention and integration into daily living.

The post-treatment questionnaire, was returned by n = 25 participants (61%) (24 completers and one non-completer). 24 of the 25 family caregivers (96%) who had responded to the questionnaire agreed or strongly agreed with the statement ‘overall, I am satisfied with OAse’. This suggests a generally high level of satisfaction with the intervention. They evaluated the intervention contents as clear and understandable, and the technical usability as good. The questionnaire responses indicated that the intervention provided practical motivations for and could be integrated in daily living and impulses for dealing with difficult situations. However, fewer than half of the participants considered the level of personal contact sufficient (Fig. 2).

The thematic analysis of the three open-ended questions revealed that large majority of the 25 participants (88%) who completed the post-treatment questionnaire experienced the fictitious caregivers guiding through the intervention as particularly helpful. The participants described that the model caregivers’ reports have made them aware that they are not alone with their situation and that their way of dealing with the situation is appropriate. In addition, the following aspects of the intervention were identified as particularly helpful: the relaxation exercise (76%), the flexibility of the online format (72%), and the usability of the intervention (56%). Reported barriers were technical problems (20%) and difficulties in integrating the intervention into their activities of daily living due to lack of time (16%). Participants stated that the intervention would be improved with the possibility of getting into contact with other family caregivers (20%) and more in-depth information on specific topics, such as coping with fear and anxiety (12%) and coping with the patient's palliative condition (8%). About one third of participants (32%) specified that they felt alone working on the intervention and would have liked more personal contact, e.g., in the form of 2–3 additional face-to-face sessions.

### Secondary outcomes

The secondary outcomes were analyzed on the basis of 25 returned post-intervention questionnaires, and we conducted a per-protocol analysis. Following correction with the Bonferroni–Holm method, the Wilcoxon signed-rank test indicated no significant changes in self-efficacy (GESE and CGI) and emotional distress (SV-POMS-D). There was a trend toward improvement in the CGI *subscale managing difficult interactions* and emotions and in the POMS subscale *depression/anxiety*. Although clinically relevant, these differences were not statistically significant (Table 2).

**Table 2:** Questionnaire scores at the baseline (T_0_) and post-intervention (T_1_).

	T_0_			T_1_			*P *(corr.)	*R*
	Med	Mean	SD	Med	Mean	SD		
GSES score	28.50	28.00	4.06	30.00	28.52	3.85	0.78	0.12
CGI score	64.00	64.05	12.57	72.00	71.26	12.27	0.06	0.39
Subscale *caring for oneself*	29.00	28.66	6.29	32.00	31.35	6.22	0.12	0.33
Subscale *managing difficult interactions and emotions*	36.00	36.08	7.47	42.00	39.91	7.48	0.06	0.38
**SV-POMS-D**								
Subscale *depression/anxiety*	28.00	27.50	16.51	16.00	20.69	17.91	0.06	0.39
Subscale *fatigue*	19.00	20.31	10.39	15.00	18.35	10.82	0.49	0.21
Subscale *hostility*	14.00	13.50	10.57	11.00	11.39	9.21	0.30	0.26
Subscale *vigor*	19.50	18.95	7.54	19.50	20.68	8.29	0.78	0.13

*P*-values from the Wilcoxon signed-rank test with Bonferroni–Holm correction.

*Med*, median; *r*, effect size (0.10—small effect, 0.30—medium effect, 0.50—large effect); *GESE*, General Self-Efficacy Scale; *CGI*, Caregiver Inventory; *SV-POMS-D*, German short version of the Profile of Mood States.

## Discussion

The present study investigated the feasibility and acceptability of self-guided Internet-based intervention for family caregivers of patients with cancer (OAse). In recent years, a small number of IBIs were developed for family caregivers of patients with cancer^42,43^. Most previous studies were either couple-based interventions focusing on communication and symptom management or interventions primarily developed for partners of cancer patients. The distinctive aspects of OAse are that the intervention is (1) specifically targeted at family caregivers addressing their unique needs and (2) not only suited for the spouses of patients but also for adult children, other family members, or peers who feel a sense of belonging to the patients. Overall, the intervention demonstrated good feasibility and high acceptability. The technological skills and private use of digital media in our sample seem representative and thus no bias in favor of participants with higher eHealth literacy should be expected. The overall completion rate was 68% and 75% of participants carried out a substantial part of the intervention (at least 4 of 6 lessons). Thus, overall adherence can be considered moderate^44^, but it is similar to other studies of telehealth interventions for family caregivers^28,30,31^. The post-treatment questionnaire showed a high overall level of family caregiver satisfaction with the intervention. The intervention was considered helpful and could be well-integrated into the activities of daily living.

The participation rate of n = 41 family caregivers appears rather low for the recruitment period of 10 months (June 2018 to April 2019). Difficulties regarding recruitment and an initially low participation rate are also reported in other studies on IBIs for family caregivers of patients with cancer^29^. IBIs are considered as promising approach to provide facilitated access to psychosocial support for family caregivers and they offer an important opportunity to reduce several common barriers of using psychosocial interventions (e.g., time constraints, restraint due to shame, or social desirability). But in fact there are serious challenges with recruitment and it seems difficult to overcome the personal constraints of family caregivers, so further research is needed to resolve participation barriers. Some recruitment barriers could be the lack of confidence in the technology or fear of time-consuming data entry^45^. Roughly two-thirds of the participants were recruited via a face-to-face conversation, while only one-third were recruited via flyers, posters, and the website. The personal approach of family caregivers seems to be of particular importance for addressing individual constraints and barriers. In their review, Adelmann et al.^17^ noted that clinicians tended to overlook the caregiving perspective and its related burdens and that family caregivers may not be accustomed to receiving attention or being addressed for psychosocial interventions. This observation is consistent with our finding that providers must be particularly aware of including family caregivers of patients with cancer in the care process.

The drop-out rate of OAse was approximately one-third. This attrition rate is in line with the results of a systematic review by Donkin et al.^46^. They concluded that high attrition and low adherence are the main limitations of online self-help interventions in cancer and non-cancer populations. There is no evidence of a selective drop-out of certain groups of family caregivers of patients with cancer and most dropouts occurred at the beginning of the intervention. Therefore, it can be assumed that most non-completers realized rather at an early stage that OAse did not fit their current needs or expectations. The reasons reported for dropping out from OAse (e.g., technical difficulties, lack of time, patient deceased during participation, and dissatisfaction with the content) correspond to the experiences of comparable feasibility studies^29,47^. The aspect of the “dosage” of a web-based intervention for family caregivers of patients with cancer is a widely discussed issue with no clear recommendations yet^48,49^. OAse was marginally structured, and only the sequence of the individual lessons was specified to enable the participants to deal with the intervention as flexibly as possible. Whether a more structured time frame for OAse (e.g., one lesson per week) or other measures to encourage participant engagement (e.g., regular email messages) would have increased the commitment and adherence to family caregivers remains to be investigated.

A distinctive feature of OAse is that the intervention addresses not only patients’ partners but also their adult children or parents. As expected most of our participants were spouses, but a considerable proportion also comprised adult children of cancer patients. In our view, it is a particularly encouraging result that we were able to include the adult children of patients because this group is underserved and understudied compared with caregiving partners^50^. The fact that adult children participated in the intervention indicates that there is a need among this target group. Further, we did not make any restrictions regarding the patient's clinical characteristics (e.g., cancer diagnosis, time since diagnosis, kind of treatment, and palliative or curative intent). This was because we wanted to reach a broad target group and avoid overlooking a small but important subgroup of family caregivers who would have benefited from the intervention. However, this was at the expense of tailoring the intervention to the individual needs of the family caregivers, which was partly reflected in their feedback.

Despite overall high satisfaction with the program, only about two-thirds of the participants reported that OAse provided them with new information. This could be because family caregivers are very concerned with the diseases and have acquired the relevant information in dealing with the disease-related circumstances. At the same time, it is also possible that OAse, due to its orientation toward a heterogeneous target group was too unspecific for some participants and, therefore, did not meet their needs and expectations. Recent studies have emphasized the importance of addressing family caregivers’ needs as accurately as possible^27^. Hence, we see the potential for improvement in terms of the individualization of the intervention, e.g., individual sequence of lessons or optional lessons on specific topics, such as treatment or entity-specific content or a lesson on coping with the palliative care condition. Another—but extensive—possibility to improve the intervention could be to develop different versions of OAse depending on the relationship to the patient (partner vs. adult children) and the stage of the disease (curative vs. palliative situation). This could allow a thematic specification and thus a better orientation towards the specific needs of the addressed target group. The main point of criticism of the participants concerned the extent of personal contact, which was rated as insufficient by slightly more than half of the participants. Nonetheless, the participants rarely used weekly telephone consultations and personal contact via the chat function. This reflects our clinical experience that family caregivers often struggle to seek psychosocial support on their initiative. Many IBIs use online support groups, which enable participants to interact with one another. Besides integrating an online support group, providing a guided intervention with individual feedback at the end of each lesson could be another way to meet participants’ needs for more personal contact.

Another important aspect is the question of the right timing for such an intervention. One consideration in this context might be that caregivers of patients with metastatic cancer in a late stage of their caregiving do not have the time and emotional resources to engage with an IBI. Some studies reveal that the prevalence of psychological morbidity among family caregivers of people with terminal cancer is alarmingly high and that family caregivers of terminal-ill patients report the need for high levels of information and psychosocial support^51–53^. Some participants in present study also expressed the desire that the issue of coping with the palliative situation should be more integrated into the intervention and that targeted information and psychosocial support should be provided. Despite the high number of very/extremely important needs and needs reported to be unmet, the number of family caregivers of patients with advanced cancer who use psychosocial support services is generally very low^54^. Therefore, an IBI could be a crucial support especially for family caregivers of people with terminal cancer to overcome the aforementioned barriers to uptake. It goes without saying that, especially in the context of an IBI, it is important to treat this issue with sensitivity and to offer the possibility of personal contact if needed.

In a recently published review, Kedia et al.^55^ demonstrated the positive effects of psychosocial interventions on caregiver burden outcomes, with the most frequently reported improvements being distress, anxiety, depression, overall quality of life, self-efficacy, and coping skills. In the present study, we did not use a control group, and the sample size was too small to detect changes in secondary outcome criteria. Nevertheless, the positive trends we found in managing difficult interactions and emotions and in the POMS subscale depression/anxiety are highly relevant for the further development and evaluation of OAse in a subsequent confirmatory trial.

### Limitations

Our study’s primary objective was to evaluate the feasibility and acceptance of the OAse intervention, so we conducted a single-arm study. The absence of the control group limits the internal validity of our research. It does not allow for a definite conclusion on the efficacy of OAse concerning the observed changes within the secondary outcome criteria. In our study sample, no restrictions were made regarding the characteristics of the family caregivers (e.g., sex, age, and relationship to the patient) or the patients (e.g., tumor entity or treatment stage). In order to avoid overlooking a small but important subgroup of family caregivers who would have benefited from the intervention, we accepted the resulting heterogeneity of our study sample. Another limitation is that the post-treatment questionnaire, which measures participant satisfaction and secondary outcomes, was returned by 61% of the participants (the majority of whom were completers). Because this was a feasibility study, the level of missing data was documented but no imputation was performed. However, this ratio is not representative of the entire sample and therefore may result in a loss of a substantial amount of information and bias the data in favor of our intervention.

## Conclusion and implication

Hitherto, specialized psychological Internet interventions for family caregivers of patients with cancer are rare. In the present study, the self-guided Internet-based intervention for family caregivers of patients with cancer (OAse) has been proven to be feasible. Our results demonstrate a general satisfaction with the intervention and indicate that OAse can be implemented well in family caregivers’ everyday lives. There were positive tendencies regarding the family caregivers’ coping strategies, especially in managing difficult interactions and emotions. Thus, the intervention could substantially improve family caregivers’ psychological condition, increasing their competency and self-efficacy. In a next step, the program will be adapted based on the qualitative feedback of the participants. Subsequently, OAse’s clinical efficacy will be tested in an evaluative research design.

## Supplementary Information

Supplementary Figure S1.

## Data Availability

The data that support the findings of this study are available from the corresponding author upon reasonable request.

## References

[1] Ugur O, Elcigil A, Arslan D, Sonmez A (2014). Responsibilities and difficulties of caregivers of cancer patients in home care. Asian Pac. J. Cancer Prevent..

[2] Lund L, Ross L, Petersen MA, Groenvold M (2014). Cancer caregiving tasks and consequences and their associations with caregiver status and the caregiver’s relationship to the patient: A survey. BMC Cancer.

[3] Brusilovskiy E, Mitstifer M, Salzer MS (2009). Perceived partner adaptation and psychosocial outcomes for newly diagnosed stage I and stage II breast cancer patients. J. Psychosoc. Oncol..

[4] Julkunen J, Gustavsson-Lilius M, Hietanen P (2009). Anger expression, partner support, and quality of life in cancer patients. J. Psychosom. Res..

[5] Applebaum AJ, Breitbart W (2013). Care for the cancer caregiver: A systematic review. Palliat. Support. Care.

[6] Zarit SH, Todd PA, Zarit JM (1986). Subjective burden of husbands and wives as caregivers: A longitudinal study. Gerontologist.

[7] Goren A, Gilloteau I, Lees M, DaCosta Dibonaventura M (2014). Quantifying the burden of informal caregiving for patients with cancer in Europe. Supportive Care Cancer.

[8] Williams AL (2018). Family caregivers to adults with cancer: The consequences of caring. Recent Results Cancer Res..

[9] Leroy T, Fournier E, Penel N, Christophe V (2016). Crossed views of burden and emotional distress of cancer patients and family caregivers during palliative care. Psychooncology.

[10] Sklenarova H (2015). Psychometric evaluation of the German version of the supportive care needs survey for partners and caregivers (SCNS-P&C-G) of cancer patients. Eur. J. Cancer Care.

[11] Selman LE (2018). Patients’ and caregivers’ needs, experiences, preferences and research priorities in spiritual care: A focus group study across nine countries. Palliat. Med..

[12] Fletcher BS, Miaskowski C, Given B, Schumacher K (2012). The cancer family caregiving experience: An updated and expanded conceptual model. Eur. J. Oncol. Nurs..

[13] Giesler, J. M. *et al.* Out-patient psychosocial cancer counseling centers and their clients—Services provided and service utilization by patients and patients’ relatives. [Ambulante psychoonkologische Versorgung durch Krebsberatungsstellen—Leistungsspektrum und Inanspruchnahme durch Patienten und Angehörige]. *Psychother. Psychosom. Med. Psychol.* **65**, 450–458. 10.1055/s-0035-1554718 (2015). 10.1055/s-0035-1554718 26200246

[14] Hartmann M, Haun M, Sklenarova H, Zimmermann-Schlegel V, Herzog W (2017). Psycho-oncological care in rural and urban areas. Experiences and desires of cancer patients and caregivers. Onkologe.

[15] Dilworth S, Higgins I, Parker V, Kelly B, Turner J (2014). Patient and health professional’s perceived barriers to the delivery of psychosocial care to adults with cancer: A systematic review. Psychooncology.

[16] Lichtenthal WG (2011). Underutilization of mental health services among bereaved caregivers with prolonged grief disorder. Psychiatr. Serv..

[17] Adelman RD, Tmanova LL, Delgado D, Dion S, Lachs M (2014). S Caregiver burden: A clinical review. JAMA.

[18] Mosher CE (2013). Support service use and interest in support services among distressed family caregivers of lung cancer patients. Psychooncology.

[19] Haun MW, Sklenarova H, Zimmermann-Schlegel V, Herzog W, Hartmann M (2018). Psycho-oncology care in rural areas: Results from a cross-sectional survey on the utilisation of community-based psychosocial support services. Bundesgesundheitsblatt Gesundheitsforschung Gesundheitsschutz.

[20] Heynsbergh N, Botti M, Heckel L, Livingston PM (2019). Caring for the person with cancer and the role of digital technology in supporting carers. Supp. Care Cancer.

[21] Agboola SO, Ju W, Elfiky A, Kvedar JC, Jethwani K (2015). The effect of technology-based interventions on pain, depression, and quality of life in patients with cancer: A systematic review of randomized controlled trials. J. Med. Internet Res..

[22] McAlpine H, Joubert L, Martin-Sanchez F, Merolli M, Drummond KJ (2015). A systematic review of types and efficacy of online interventions for cancer patients. Patient Educ. Couns..

[23] Slev VN (2016). Effects of eHealth for patients and informal caregivers confronted with cancer: A meta-review. Int. J. Med. Inform..

[24] Badr H (2016). Development and usability testing of a web-based self-management intervention for oral cancer survivors and their family caregivers. Eur. J. Cancer Care.

[25] Northouse L (2014). A tailored web-based psychoeducational intervention for cancer patients and their family caregivers. Cancer Nurs..

[26] Benson JJ (2020). Online social support groups for informal caregivers of hospice patients with cancer. Eur. J. Oncol. Nurs..

[27] Leow MQ, Chan SW (2016). Evaluation of a video, telephone follow-ups, and an online forum as components of a psychoeducational intervention for caregivers of persons with advanced cancer. Palliat. Supp. Care.

[28] Köhle N (2021). Web-based self-help intervention for partners of cancer patients based on acceptance and commitment therapy and self-compassion training: A randomized controlled trial with automated versus personal feedback. Supp. Care Cancer.

[29] Schuit AS, Holtmaat K, Hooghiemstra N, Jansen F, Lissenberg-Witte BI (2020). Efficacy and cost-utility of the eHealth self-management application ‘Oncokompas’, helping partners of patients with incurable cancer to identify their unmet supportive care needs and to take actions to meet their needs: A study protocol of a randomized controlled trial. Trials.

[30] Scott K, Beatty L (2013). Feasibility study of a self-guided cognitive behaviour therapy internet intervention for cancer carers. Aust. J. Prim. Health.

[31] Bodschwinna D (2022). A psycho-oncological online intervention supporting partners of patients with cancer (PartnerCARE): Results from a randomized controlled feasibility trial. Psychooncology.

[32] Marzorati C, Renzi C, Russell-Edu SW, Pravettoni G (2018). Telemedicine use among caregivers of cancer patients: Systematic review. J. Med. Internet Res..

[33] Antonovsky, A. *Unraveling the Mystery of Health—How People Manage Stress and Stay Well*. (San Francisco, 1987).

[34] Romito F, Goldzweig G, Cormio C, Hagedoorn M, Andersen BL (2013). Informal caregiving for cancer patients. Cancer.

[35] Eldredge LKB, Markham CM, Ruiter RA, Fernández ME, Kok G, Parcel GS (2016). Planning Health Promotion Programs: An Intervention Mapping Approach.

[36] Braun V, Clarke V (2006). Using thematic analysis in psychology. Qual. Res. Psychol..

[37] Schwarzer, R. & Jerusalem, M. Generalized self-efficacy scale. in *Measures in Health Psychology: A User’s Portfolio. Causal and Control Beliefs *(eds. Weinman, J., Wright, S.S., Johnston, M.). 35–37 (Windsor, 1995).

[38] Merluzzi TV, Philip EJ, Vachon DO, Heitzmann CA (2011). Assessment of self-efficacy for caregiving: The critical role of self-care in caregiver stress and burden. Palliat. Supp. Care.

[39] Albani C (2005). The German short version of “Profile of Mood States“ (POMS): Psychometric evaluation in a representative sample. Psychother. Psychosom. Med. Psychol..

[40] Holm S (1979). A simple sequentially rejective multiple test procedure. Scand. J. St..

[41] Rosenthal, R. Parametric measures of effect size. in *The Handbook of Research Synthesis* (eds. Cooper, H., Hedges, L. V.). 231–244 (1994).

[42] Marzorati C, Renzi C, Russell-Edu SW, Pravettoni G (2018). Telemedicine use among caregivers of cancer patients: Systematic review. J. Med. Internet. Res..

[43] Shin JY, Kang TI, Noll RB, Choi SW (2018). Supporting caregivers of patients with cancer: A summary of technology-mediated interventions and future directions. Am. Soc. Clin. Oncol..

[44] Ownsworth T, Chan RJ, Jones S, Robertson J, Pinkham MB (2021). Use of telehealth platforms for delivering supportive care to adults with primary brain tumors and their family caregivers: A systematic review. Psychooncology.

[45] Nemecek R (2019). Telemedically augmented palliative care. Wien. Klein. Wochenschr..

[46] Donkin L (2011). A systematic review of the impact of adherence on the effectiveness of e-therapies. J. Med. Internet. Res..

[47] DuBenske LL (2014). CHESS improves cancer caregivers’ burden and mood: Results of an eHealth RCT. Health Psychol..

[48] Heynsbergh N, Heckel L, Botti M, Livingston PM (2018). Feasibility, useability and acceptability of technology-based interventions for informal cancer carers: A systematic review. BMC Cancer.

[49] Tang WP, Chan CW, So WK, Leung DY (2014). Web-based interventions for caregivers of cancer patients: A review of literatures. Asia-Pac. J. Oncol. Nurs..

[50] Lambert SD (2012). The unmet needs of partners and caregivers of adults diagnosed with cancer: A systematic review. BMJ Supp. Palliat. Care.

[51] Areia NP, Fonseca G, Major SDO, Relvas AP (2019). Psychological morbidity in family caregivers of people living with terminal cancer: Prevalence and predictors. Palliat. Supp. Care.

[52] Aoun SM, Kristjanson LJ, Hudson PL, Currow DC, Rosenberg JP (2005). The experience of supporting a dying relative: Reflections of caregivers. Prog. Palliat. Care.

[53] Andershed B (2006). Relatives in end-of-life care–part 1: A systematic review of the literature the five last years, January 1999–February 2004. J. Clin. Nurs..

[54] Ullrich, A. *et al.* Supportive care needs and service use during palliative care in: A prospective longitudinal study. *Supp. Care Cancer* **29**, 1303–1315. 10.1007/s00520-020-05565-z (2021) 10.1007/s00520-020-05565-z PMC7843549 32632761

[55] Kedia SK (2020). Psychosocial interventions for informal caregivers of lung cancer patients: A systematic review. Psychooncology.

