# Supplementary material for: Feasibility study of a self-guided internet-based intervention for family caregivers of patients with cancer (OAse)
Source: Sci Rep. 2022 Oct 6;12:16713. doi: 10.1038/s41598-022-21157-9 (PMC9537301; doi:10.1038/s41598-022-21157-9)
Supplement: Supplementary file 1 — Supplementary Figure S1. [file 41598_2022_21157_MOESM1_ESM.docx]

**Feasibility Study of a self-guided internet-based intervention for family caregivers of patients with cancer (OAse)**

Miriam Grappa, Johanna Ell, Senta Kiermeier, Markus W. Haun, Andrea Kübler, Hans-Christoph Friederich, Imad Maatouk*

**Supplementary Figure (S1).** Two extracts (Screenshots) of Lesson 3 of the OAse-Intervention (“One’s own feelings – understanding and regulating”). Since OAse is a German language intervention, the texts in the figure were translated by the authors for this publication.


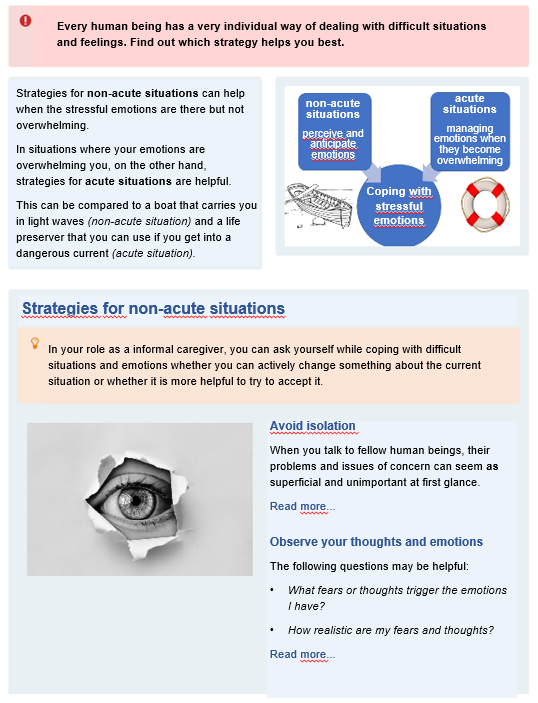


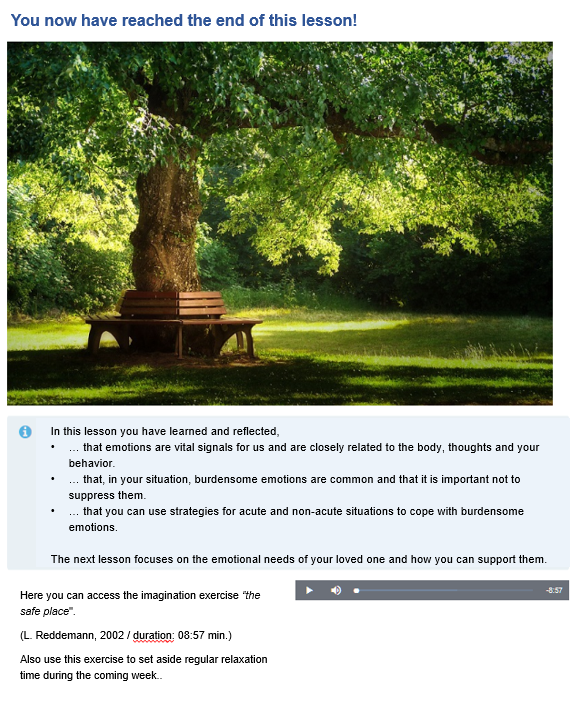


*Corresponding author:

Imad Maatouk

Department of General Internal and Psychosomatic Medicine, University Clinic Heidelberg, Im Neuenheimer Feld 410, 69120 Heidelberg, Germany.

Section of Psychosomatic Medicine, Psychotherapy and Psychooncology, Department of Internal Medicine II, Julius-Maximilian University Würzburg, Würzburg, Germany.

email: Maatouk_I@ukw.de
